# Research advances in the etiology of in-stent restenosis of coronary arteries

**DOI:** 10.3389/fcvm.2025.1585102

**Published:** 2025-07-18

**Authors:** Hangrui Bai, Bin Zhang, Yurong Sun, Xinran Wang, Bo Luan, Xiaojiao Zhang

**Affiliations:** ^1^ People's Hospital, China Medical University, Shenyang, China; ^2^ Cardiovascular Disease Treatment Center, The People's Hospital of Liaoning Province, Shenyang, China; ^3^ Graduate School of Dalian Medical University, Dalian, China

**Keywords:** coronary atherosclerotic heart disease, percutaneous coronary intervention, coronary artery stent, in-stent restenosis, etiological study

## Abstract

This article conducts a systematic literature analysis, focusing on exploring the etiological factors of coronary in—stent restenosis(ISR). It is found that the occurrence of ISR is influenced by a variety of factors, including patient—related biological factors (such as diabetes and smoking), lesion anatomical factors (such as calcification and lesion length), and factors related to the surgical procedure (such as incomplete stent expansion and stent malposition). Regarding the prevention of coronary in—stent restenosis, individualized treatment strategies need to be developed by combining patient characteristics, lesion anatomy, and surgical techniques.

## 1 Background

In-stent restenosis (ISR) is a key challenge affecting patient prognosis after percutaneous coronary intervention (PCI). Although the application of drug-eluting stents (DES) has significantly reduced the incidence of ISR, its pathophysiological mechanisms are complex and involve the interplay of multiple factors.

## 2 Main text

Since the advent of percutaneous coronary intervention (PCI) technology, in-stent restenosis (ISR) has posed a significant challenge to the clinical efficacy of PCI. Despite the widespread adoption of drug-eluting stents (DES), advancements in the science of coronary stent materials, and the optimization of clinical medication regimens, the incidence of ISR in the first year following PCI has markedly decreased from 20%–30% during the bare-metal stent (BMS) era to 5%–10% (1). Nevertheless, ISR remains the leading cause of stent failure and the most common indication for repeat coronary revascularization, with approximately 25% of patients presenting with acute myocardial infarction (AMI) (2). Notably, compared to PCI patients without ISR, both the incidence and mortality rates are significantly elevated in those with ISR (3). Therefore, a thorough exploration of the etiological mechanisms underlying ISR, along with the development of individualized treatment plans based on a comprehensive assessment of patient characteristics and lesion features, is of great clinical significance for enhancing patient outcomes.

Traditionally, the ISR is based on the visual assessment of coronary angiography (CAG), which means that a new lesion appears within the stent or within 5 mm of the stent edge, and the diameter stenosis is ≥50% (4). However, in clinical practice, the definition of ISR is more stringent: in addition to the stenosis shown by CAG, patients must also have ischemic symptoms or a blood flow reserve fraction <0.80; even in the absence of symptoms and signs of myocardial ischemia, a reduction in lumen diameter of 70% or more can also be considered as ISR. This comprehensive definition based on ischemic symptoms, blood flow reserve fraction, and stenosis degree not only helps to more accurately assess the clinical significance of restenosis but also provides a basis for the development of individualized treatment plans (5).

Traditional coronary angiography, while capable of diagnosing ISR, fails to differentiate the underlying pathological heterogeneity of restenosis due to its two-dimensional perspective. However, the advent of intravascular imaging and histopathological research has elucidated the heterogeneous nature of ISR—distinct classifications exhibit significant divergence in pathogenesis, treatment response, and prognosis. To systematically guide clinical decision-making, contemporary ISR classification integrates a tripartite standard encompassing angiographic morphology, tissue composition characteristics, and imaging biomarkers (Table 1). This classification framework not only delineates the clinical definitions and features of ISR but also elucidates their therapeutic implications, thereby providing an objective foundation for precision interventions (4, 6, 7).

**Table 1:** Current classification systems for in-stent restenosis (ISR).

Classification basis	Type	Definition and features	Clinical implications
Angiographic classification	Type I: focal	Stenosis confined within the stent or margins (length ≤10 mm)	Best prognosis; drug-coated balloon (DCB) efficacy >90%
	Type II: diffuse	Stenosis extending beyond focal margins but confined within the stent (length >10 mm)	Requires intravascular imaging assessment; DCB or new stent implantation
	Type III: proliferative	Diffuse stenosis extending beyond stent borders (length >10 mm)	High restenosis risk; often requires atherectomy + new stent
	Type IV: total occlusion	100% stent occlusion	Worst prognosis; requires mechanical recanalization or bypass surgery
Histopathological classification	Neointimal hyperplasia	Dominant smooth muscle cell proliferation with low lipid content (OCT: homogeneous high signal)	Responsive to antiproliferative agents (e.g., paclitaxel)
	Neoatherosclerosis	Foam cell/cholesterol crystal accumulation with thin-cap or calcification (OCT: low-signal lipid core)	High late thrombosis risk; requires intensive lipid-lowering + anti-inflammatory therapy
Intravascular imaging classification	Calcified pattern	Calcium arc >180° on OCT or calcium thickness >0.5 mm on IVUS	Requires plaque modification (atherectomy/shockwave balloon)
	Stent underexpansion	Minimal stent area (MSA) <4.5 mm² or expansion ratio <80%	Post-dilation optimization critical
	Stent fracture	Discontinuity of stent struts on IVUS/OCT	Requires bioresorbable scaffold or surgical intervention

**Figure 1:**
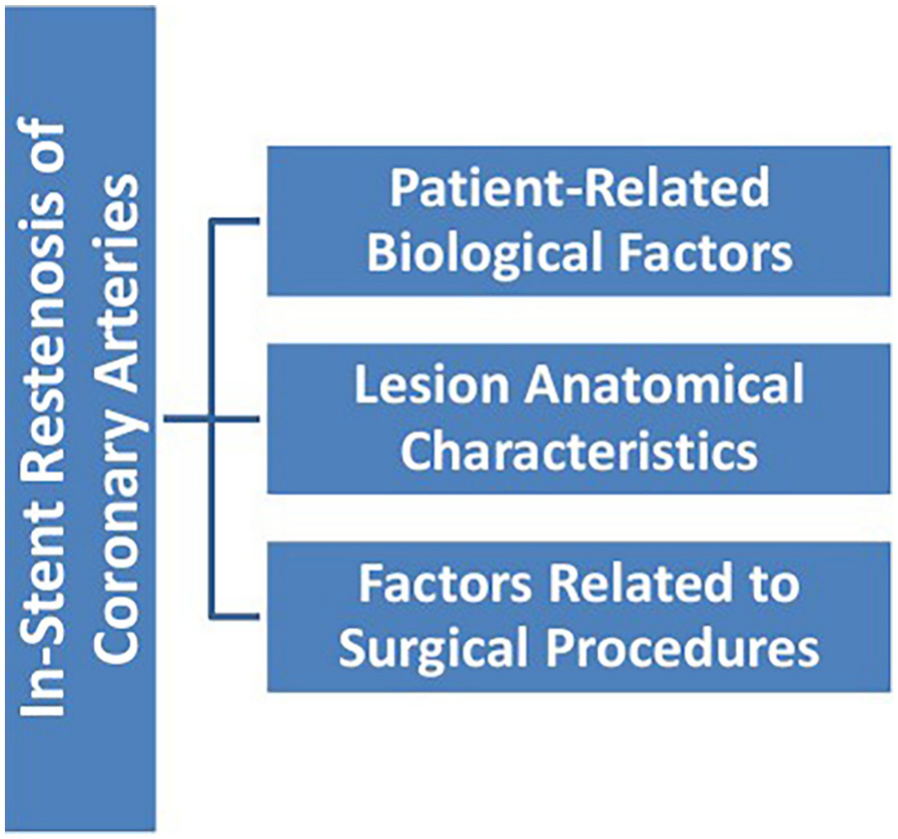
The mechanisms of ISR.

**Figure 2:**
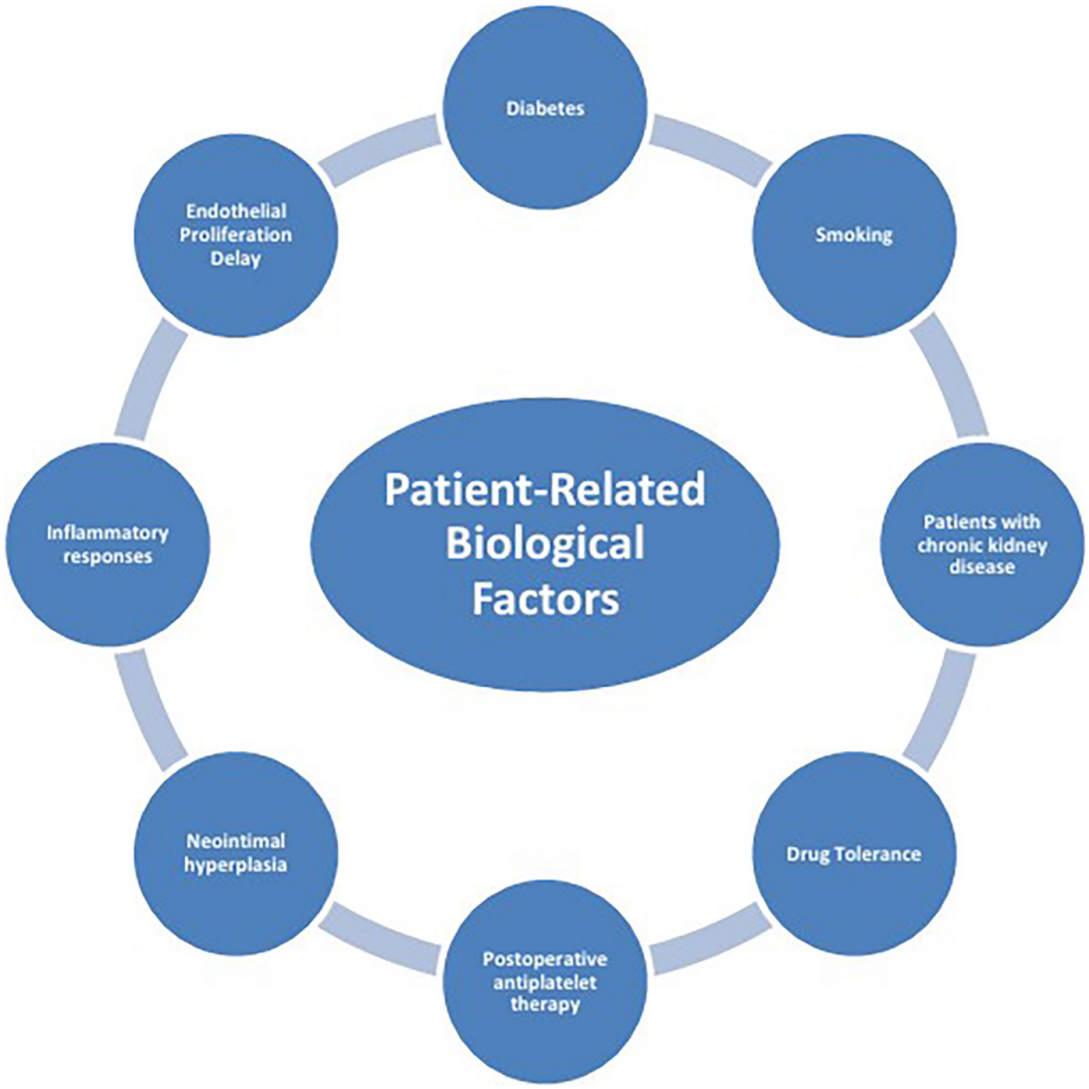
Patient-Related biological factors.

**Figure 3:**
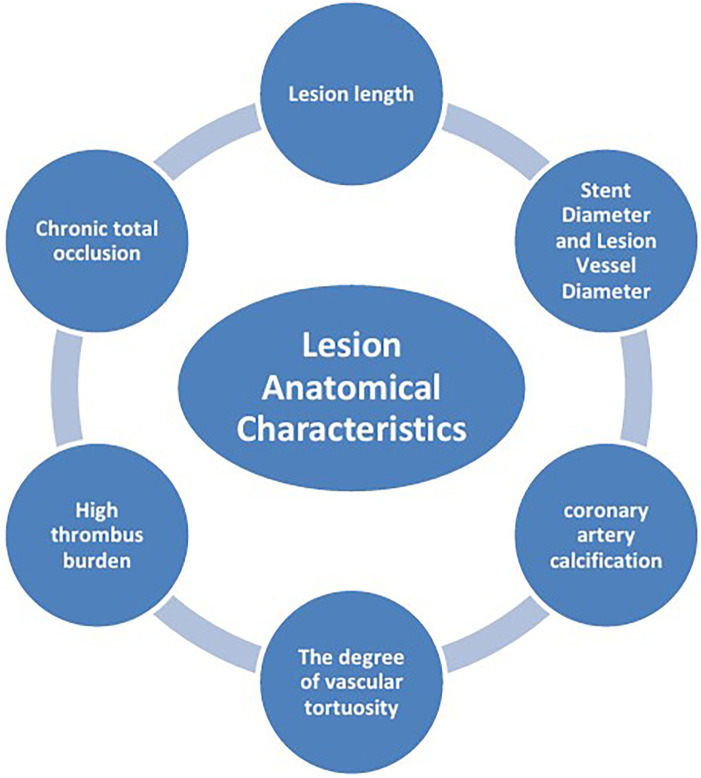
Lesion anatomical characteristics.

**Figure 4:**
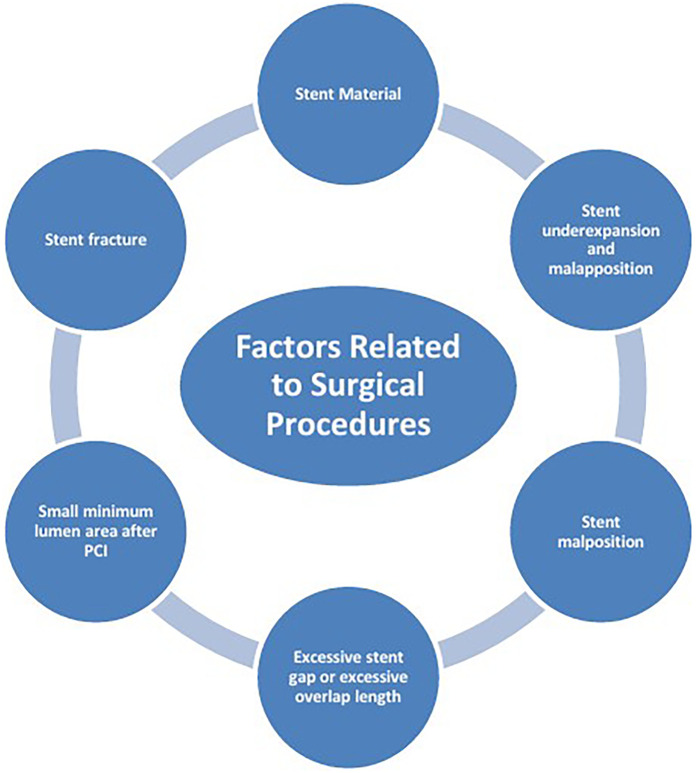
Factors related to surgical procedures.

The mechanisms of ISR involve the interaction of multiple factors (Figure 1), mainly including patient-related biological factors (Figure 2), lesion anatomical characteristics (Figure 3), and factors related to the surgical procedure (Figure 4).

### 2.1 Patient-related biological factors

#### 2.1.1 Diabetes

Diabetes is an important independent risk factor for ISR. The endothelial dysfunction that is common in diabetic patients not only accelerates the process of atherosclerosis but also significantly increases the risk of ISR. Studies (8) have shown that the neointimal hyperplasia of the coronary arteries in diabetic patients is more pronounced, and the mechanism may be related to a hyperglycemic environment promoting the proliferation and migration of coronary artery smooth muscle cells (9). A recent large-scale cohort study from Sweden has delved into the correlation between glycemic control levels in patients with type 2 diabetes mellitus and the risk of stent failure (10). A recent large-scale cohort study from Sweden, which included 52,457 patients with type 2 diabetes mellitus who had undergone implantation of second-generation drug-eluting stents nationwide between 2010 and 2020, stratified glycemic control levels using a reference group with an HbA1c of 6.1%–7.0%. The primary endpoint of the study was stent failure, including in-stent restenosis and stent thrombosis. The results showed that the risk of stent failure increased in a dose-dependent manner with the elevation of HbA1c levels: when HbA1c was ≥10.1%, the risk of stent failure was significantly increased by 33% compared with the reference group (*p* < 0.01). The results of sensitivity analysis further supported this conclusion, confirming that poor glycemic control is significantly associated with an increased risk of stent failure due to ISR.

Furthermore, substantial evidence indicates that diabetes modulates the expression of inflammatory cytokines (11, 12), thereby potentially influencing the pathogenesis of ISR (11). Specifically, the upregulation of pro-inflammatory factors (such as IL-6 and TNF-α) in the bodies of diabetic patients may exacerbate the inflammatory response of the vascular wall, thereby promoting the occurrence of restenosis (13).

#### 2.1.2 Smoking

Smoking, an established risk factor for cardiovascular disease, has been widely confirmed as a strong independent predictor for ISR. Its pathogenic mechanisms mainly involve the activation of oxidative stress and inflammatory responses. Studies have shown that smoking can induce the formation of a pro-inflammatory microenvironment in the arterial intima, characterized by a significant increase in the levels of inflammatory mediators. These pathophysiological changes not only accelerate the process of neointimal hyperplasia but also, by promoting platelet activation and fibrin deposition, lead to an increased tendency for thrombosis, thereby further impairing the vessel's repair capacity (14).

#### 2.1.3 Patients with chronic kidney disease (CKD)

Research conclusions show that the incidence of ISR in patients with chronic kidney disease (CKD) is significantly higher than that in individuals with normal renal function (*p* < 0.01) (15). From a pathophysiological perspective, this is mainly attributed to the systemic inflammatory response and vascular calcification associated with CKD (16). The impact of CKD on the local microenvironment of stents is mainly reflected in the exacerbation of intimal hyperplasia and inflammation—mediated vascular remodeling (17). These pathological changes are all important promoters of ISR. The systemic chronic inflammatory state unique to CKD patients significantly increases the risk of ISR.

A retrospective study from China (18) analyzed 164 CKD patients who underwent coronary stent implantation between 2017 and 2022. The study found that the CHA2DS2-VASc score, fibrinogen (FIB) levels, and the neutrophil-to-lymphocyte ratio (NLR) are independent predictors of ISR. Notably, the combined predictive power of these three indicators is significant, with the area under the ROC curve (AUC) reaching 0.797 (95% CI: 0.732–0.862), indicating that their combination can effectively predict the occurrence of ISR in CKD patients. The sample size of this retrospective study is limited (*n* = 164), and future multi-center prospective studies are needed to verify the conclusions.

It is worth noting that CKD patients often have multiple cardiovascular diseases, which not only increase the risk of ISR but also significantly raise the complexity of clinical treatment and the incidence of adverse events (19).

#### 2.1.4 Drug tolerance

Some patients may exhibit high on-treatment platelet reactivity (HPR) after antiplatelet therapy, a phenomenon that can significantly reduce the clinical efficacy of antiplatelet treatmentand (20), in turn, increase the risk of ISR. Studies have shown that the occurrence of HPR is closely related to genetic factors, especially the polymorphism of the P2Y12 receptor gene (21), This genetic susceptibility not only limits the choice of postoperative antiplatelet treatment options but may also increase the risk of ISR by affecting the effectiveness of platelet inhibition.

#### 2.1.5 Postoperative antiplatelet therapy

Postoperative antiplatelet therapy is a cornerstone strategy for preventing ISR. As a key measure to prevent local thrombosis and systemic ischemic events after PCI, antiplatelet therapy reduces the risk of ischemia while also increasing the incidence of bleeding complications (22). With the innovation of stent technology and the in-depth understanding of the mechanisms of ischemia recurrence after PCI, the antiplatelet treatment strategy has undergone significant evolution over the past few decades. In particular, dual antiplatelet therapy (DAPT), as the main strategy for preventing thrombosis after PCI, has been widely used in clinical practice for nearly 30 years. DAPT can significantly reduce the risk of stent thrombosis, especially in the acute and subacute phases after PCI (23).

#### 2.1.6 Neointimal hyperplasia

Neointimal hyperplasia is the core pathophysiological mechanism of ISR. The underlying mechanisms of the development of neoatherosclerosis are not yet clear. Neoatherosclerosis may occur months to years after PCI, while atherosclerosis of the native coronary artery takes decades to develop. In-stent neoatherosclerosis (NA) has become an important clinical issue after PCI. ISR and stent thrombosis are the two major complications after coronary stent placement, which seriously affect patient prognosis. As a common pathological basis for these two complications, NA plaques are different from natural atherosclerotic plaques and usually grow around residual oxidized lipids and stent struts. The main component is foam cells formed by vascular smooth muscle cells (VSMCs) engulfing oxidized lipids at the sites of lipid residues (24). Histologically, it is characterized by the accumulation of lipid-rich foamy macrophages within the neointima, with or without a necrotic core and/or calcification (7). Stent implantation can cause vascular injury and local blood flow disturbances related to endothelial dysfunction, leading to the activation of inflammatory cells, increased thrombosis, and reduced β-lipoprotein efflux, followed by the accumulation of lipoproteins within the neointima. Immature endothelial cells with increased permeability also promote the migration of monocytes (25). Underlying native atherosclerotic plaques may also be a cause of the pathogenesis of NA. However, early pathological reports did not describe the anatomical correlation with the original atherosclerotic tissue (26).

#### 2.1.7 Inflammatory responses

Inflammatory responses significantly affect the development of ISR in both the acute and chronic phases after stent implantation. The mechanical injury to the vascular wall caused by stent implantation can trigger a local inflammatory response, recruiting inflammatory cells such as macrophages and neutrophils to the site of injury. These cells release inflammatory mediators such as tumor necrosis factor-α (TNF-α) and interleukin-1β (IL-1β), which VSMCs and promote their proliferation and migration, ultimately leading to neointimal hyperplasia (27). “Chronic inflammatory responses and endothelial dysfunction can promote the deposition of lipoproteins in the intima, accelerating the formation of NA. Studies have shown that the incidence of NA in first—generation DES is significantly higher than that in BMS. Specifically, the incidence of NA in first—generation DES is 35%, while it is 10% in BMS (27). High—sensitivity C—reactive protein (hs—CRP) and interleukin—6 (IL—6) are inflammatory markers that are closely related to the risk of ISR. A large—scale cohort study showed that the risk of major adverse cardiovascular events (MACE) in patients in the highest quartile of hs—CRP levels was 90% higher than that in the lowest quartile (aHR 1.90, 95% CI: 1.39–2.59; *P* < 0.001) (28). Therefore, monitoring inflammatory markers such as hs—CRP helps identify patients at high risk of ISR and guide the development of individualized treatment strategies. Given the key role of inflammatory responses in ISR, anti—inflammatory treatment may emerge as a new strategy for preventing ISR (28).

Some studies have confirmed that anti-inflammatory drugs such as statins and colchicine can significantly reduce hs-CRP levels and decrease the incidence of ISR (29).

#### 2.1.8 Endothelial proliferation delay

After stent implantation, the timely proliferation and migration of vascular endothelial cells are crucial for achieving complete endothelialization. Incomplete or delayed endothelialization can lead to platelet aggregation and thrombosis, significantly increasing the risk of ISR (30). Under physiological conditions, endothelial cells can rapidly cover the damaged vascular wall after stent implantation, forming a functional endothelial layer, which inhibits the migration of smooth muscle cells and thrombosis. However, factors such as mechanical injury, chronic inflammatory response, and anti—proliferative drugs released by DES may interfere with the endothelial repair process, leading to delayed endothelialization (>3 months), which in turn promotes the occurrence of ISR. Bioresorbable vascular scaffolds (BVS) provide a new approach to solving this problem. BVS can gradually degrade after completing the vascular support function (usually 6–12 months), avoiding the chronic inflammatory response and endothelial dysfunction caused by the long—term retention of traditional metal stents (31).

### 2.2 Lesion anatomical characteristics

#### 2.2.1 Lesion length

Lesion length is significantly associated with the incidence of ISR. Multiple studies have shown that both longer lesion length and stent length are associated with a higher risk of ISR. A angiographic follow-up study involving 1,181 patients (32) showed that in 87% of the cases, the stent length exceeded the lesion length (mean lesion length: 12.4 ± 6.3 mm; mean stent length: 20.0 ± 7.9 mm; mean difference: 7.6 ± 7.9 mm). During the follow-up period of 6–9 months, the mean percentage of diameter stenosis was 39.1% ± 20.1%. The results of multivariate regression analysis showed that for every 10 mm increase in in—stent lesion length, the absolute value of percentage diameter stenosis increased by 7.7% (*p* < 0.0001); and for every 10 mm increase in stent length, it independently increased the percentage diameter stenosis by 4.0% (*p* < 0.0001). In addition, the increase in stent length was significantly associated with the risk of target lesion revascularization at 9 months (odds ratio: 1.12; 95% CI: 1.02–1.24).

#### 2.2.2 Stent diameter and lesion vessel diameter

Vessel diameter is a key anatomical factor affecting ISR and it is also an important predictor of ISR after BMS and DES implantation (33). Studies have shown that smaller vessel diameter is significantly associated with a higher incidence of ISR, mainly due to the fact that in small—diameter vessels, stents are more prone to mechanical complications such as incomplete expansion and malapposition.

A secondary retrospective analysis involving 2,522 PCI patients showed that a larger stent diameter was an independent protective factor for target vessel revascularization (TVR), especially in the delayed PCI subgroup (34). It is worth noting that this ‘bigger is better’ protective effect is particularly significant in stents with diameters ranging from 2.5 to 2.9 mm (*p* < 0.01). For the treatment strategy of small—diameter vessel lesions, it is recommended to use high—resolution intravascular imaging techniques, such as optical coherence tomography (OCT). OCT can provide accurate measurements of the vessel lumen and assessment of stent apposition, which helps to optimize the surgical outcome and thus reduce the incidence of ISR (35).

#### 2.2.3 Coronary artery calcification (CAC)

Coronary artery calcified lesions are one of the major technical challenges faced by PCI. Calcified lesions not only increase the risk of incomplete stent expansion and positioning deviation during the procedure but are also closely associated with the occurrence of ISR after surgery (36). A study exploring the correlation between CAC and ISR showed that CAC is an independent predictor of ISR, and patients with severe calcified lesions had a significantly increased risk of ISR and MACE (*p* < 0.01) (37). Therefore, adequate plaque pre—treatment before stent implantation in calcified lesions is crucial. Adequate pre—treatment can ensure optimal stent apposition and long—term patency, with specific strategies including the use of cutting balloons, rotational atherectomy, or shock wave balloons, etc.

#### 2.2.4 The degree of vascular tortuosity

The degree of vascular tortuosity is one of the key anatomical factors affecting stent implantation outcomes. Highly tortuous vessels may not only impede wire passage and affect the precise positioning of the catheter and stent but also lead to incomplete stent expansion, malapposition, and even cause mechanical deformation or displacement, thereby significantly increasing the risk of ISR (38).

A study exploring the relationship between coronary artery tortuosity (CAT) and clinical outcomes indicated that CAT is an independent predictor of early and late MACE. During long—term follow—up, the incidence of ISR and TVR in patients with tortuous lesions was significantly higher than that in patients without tortuous lesions (*p* < 0.01) (39). Another study further analyzed the correlation between the severity of CAT and in—stent stenosis. The results showed that in patients with stable and unstable angina, the severity of CAT was significantly positively correlated with in—stent stenosis (40).

#### 2.2.5 High thrombus burden

High thrombus burden is an important pathological factor affecting ISR, influencing the entire process from pre—to post—surgery. In patients with acute coronary syndrome (ACS), a high thrombus burden may lead to incomplete stent expansion during the procedure and early stent thrombosis after surgery. This is because large—volume thrombi are difficult to completely remove through routine thrombus aspiration or thrombolytic therapy, and residual thrombus can significantly affect stent apposition quality and expansion effect (41). A study exploring the correlation between high thrombus burden and stent thrombosis indicated that a high thrombus burden is an independent risk factor for stent thrombosis and can increase the risk of ISR (42).

#### 2.2.6 Chronic total occlusion (CTO)

Chronic total occlusion (CTO) of coronary atherosclerosis is the most challenging subtype in the treatment of coronary artery disease, which significantly increases the risk of ISR. CTO lesions are usually accompanied by severe fibrosis and calcification, which not only increase the technical difficulty of vascular recanalization and stent implantation but also may lead to an increased incidence of ISR after surgery (43). A study exploring the correlation between coronary collateral circulation and ISR used the Rentrop grading system to assess the degree of collateral circulation. The results showed that although good collateral circulation (Rentrop grades 2–3) was more common in right coronary artery occlusion, and poor collateral circulation (Rentrop grades 0–1) was more often seen in left anterior descending artery occlusion, there was no significant difference in ISR incidence between the two groups during an average follow—up of 18 months (12.7% vs. 20.2%, *p* = 0.148). Multivariate regression analysis indicated that age, history of diabetes, and reference CTO vessel diameter were independent predictors of ISR, while the Rentrop collateral grade was not significantly associated with ISR (44).

### 2.3 Factors related to surgical procedures

#### 2.3.1 Stent material

Studies have shown that there is a significant correlation between stent material selection and the incidence of ISR. The metal stents used in the early days were mainly made of materials such as stainless steel and cobalt-chromium alloy. These materials may cause local inflammatory responses and subacute thrombosis due to mechanical injury and sustained vascular wall expansion after implantation (45).

With the advancement of material science, the development of stent surface modification techniques (such as coating treatment) has significantly improved stent performance. Surface modification can enhance the anticoagulant properties of the material, effectively reducing platelet adsorption and aggregation, thereby lowering the incidence of ISR (46). Among them, DES can specifically inhibit the proliferation and migration of VSMCs by coating the stent surface with anti—proliferative drugs (such as sirolimus or paclitaxel), thereby reducing neointimal hyperplasia and significantly lowering the risk of ISR (1).

#### 2.3.2 Stent underexpansion and malapposition

Stent underexpansion and malapposition are important surgically related factors for ISR. Stent underexpansion is common in areas with severe calcification or tortuous vessels. These anatomical features restrict the complete expansion of the stent, leading to local hemodynamic abnormalities. In clinical practice, stent underexpansion is often caused by vascular calcification or high plaque burden. It is worth noting that stent malapposition often coexists with underexpansion, and both can significantly increase the risk of ISR. Intravascular imaging techniques, such as OCT, can provide real-time assessment of stent expansion, guiding the selection of appropriate stent types and expansion pressures during the procedure. OCT can offer detailed images of the vessel lumen and stent struts, allowing for precise evaluation of stent expansion and apposition. If underexpansion is detected, the operator can use the information to adjust the stent size or apply higher expansion pressures to ensure optimal stent deployment. This real-time feedback helps to minimize the risk of ISR by addressing potential issues immediately during the surgery (47).

A prospective multicenter registry study analyzed the OCT imaging characteristics of 297 patients who presented with coronary artery syndrome related to ISR (48). All patients underwent OCT assessment before intervention, which included ISR phenotype, stent layer number, and stent expansion status. The study found that 57% of patients had neoatherosclerosis (of which 52% were calcified), 43% had neointimal hyperplasia (of which 58% were homogeneous), 43% had stent underexpansion (minimum stent area <4.5 mm^2^), and 30% had multiple stent layers. OCT assessment changed the treatment strategy in 30% of the cases. The final treatment approaches included DES implantation (61.6%) and drug-eluting balloon (DEB) angioplasty (36.1%), but only 63.2% of the patients achieved optimal treatment outcomes. At 1—year follow—up, the target vessel failure rate was 11% (95% CI, 9%–13%). Notably, stent underexpansion was associated with a significantly higher target vessel failure rate [19% [95% CI, 14%–24%] vs. 7% [95% CI, 5%–9%], *P* = 0.01]. Studies have confirmed that OCT can effectively identify neoatherosclerosis and neointimal hyperplasia, and that stent underexpansion is relatively common and associated with adverse clinical outcomes.

Long—term follow—up studies have further confirmed that stent underexpansion significantly increases the risk of ISR and TVR. Compared with the group with good stent expansion, the long—term incidence of ISR and TVR in the underexpanded group was significantly higher (*P* < 0.01) (49).

#### 2.3.3 Stent malposition

Stent malposition is a common surgical—related complication leading to ISR, often caused by imprecise stent implantation procedures or improper use of equipment during surgery. Stent malposition can result in local intimal mechanical injury, triggering a persistent inflammatory response, which in turn promotes neointimal hyperplasia and the occurrence of ISR (50). Intravascular ultrasound (IVUS) and OCT are intravascular imaging techniques that can provide real—time and accurate stent positioning monitoring. Studies have shown that stent implantation guided by IVUS/OCT can significantly reduce the incidence of stent malposition (51). These technologies provide high—resolution cross—sectional images of the blood vessels, helping the operators to promptly identify and correct stent malposition.

#### 2.3.4 Excessive stent gap or excessive overlap length

Stent gaps or overlaps are common technical challenges in multi—stent implantation procedures, mainly related to the operator's lack of experience or misjudgment during the surgery. These technical issues can significantly affect local hemodynamic characteristics: excessive stent gaps can lead to blood flow disturbances and vortex formation, increasing the risk of thrombosis; while excessive stent overlap may trigger an excessive intimal hyperplasia response (52), thereby causing stent lumen narrowing.

#### 2.3.5 Small minimum lumen area after PCI

The minimum lumen area (MLA) after surgery is one of the key indicators for evaluating the effectiveness of PCI. Inadequate MLA can lead to restricted blood flow and accelerate the progression of ISR. Clinical studies have shown that ideal stent implantation should achieve an expansion close to 100% of the original vessel diameter, and even a slight overexpansion (recommended range: 100%–110%) may be appropriate to obtain the best clinical outcomes (53).

#### 2.3.6 Stent fracture

Stent fracture is one of the important mechanical complications of ISR, which often occurs in areas of stress concentration (such as vascular flexion) (54). The fracture of the stent under abnormal shear force or excessive expansion force not only may induce acute stent—thrombosis but also carries the risk of perforating the vessel wall, leading to serious clinical consequences (55).

Intravascular imaging (IVUS/OCT) has revolutionized percutaneous coronary intervention by overcoming the limitations of conventional angiography and enabling precision guidance. The landmark OCTIVUS trial (56) established these modalities as the gold standard for complex PCI, demonstrating that meticulous correction of suboptimal stent deployment significantly reduces risks of restenosis and stent thrombosis.

## 3 Conclusion

ISR is a key issue affecting clinical outcomes after PCI. Its occurrence mechanism involves the complex interplay of multiple factors, including patient biological characteristics, lesion anatomical structure, and surgical procedures. Among patient—related factors, diabetes, smoking, and chronic kidney disease significantly increase the risk of ISR through inflammatory responses, endothelial dysfunction, and metabolic abnormalities; lesion anatomical characteristics such as long lesion length, small vessel diameter, calcification, and vascular tortuosity promote ISR occurrence through mechanical stress, hemodynamic changes, and neointimal hyperplasia; Among surgical—related factors, the choice of stent material, insufficient expansion, poor apposition, and technical defects in the procedure directly affect the long—term patency of the stent. Although the widespread use of DES and the advancement of intravascular imaging technology have significantly reduced the incidence of ISR, individualized intervention strategies for high—risk patients and complex lesions still need further optimization.

Future research should focus on the following directions: delve into the molecular mechanisms of ISR, especially the regulatory network of inflammatory signaling pathways and neointimal hyperplasia; develop new types of biodegradable stents and targeted anti—proliferative drugs to balance the contradiction between endothelial repair and excessive proliferation; promote the clinical application of intravascular imaging techniques (such as OCT and IVUS) to achieve precise stent implantation and postoperative evaluation; establish risk prediction models based on multi—omics data to formulate stratified management strategies for high—risk patients. Through multi—disciplinary collaboration and technological innovation, it is expected to improve the prevention and treatment effects of ISR while further promoting the development of the field of coronary heart disease interventional treatment.
